# Post-Surgical Analgesia in Rainbow Trout: Is Reduced Cardioventilatory Activity a Sign of Improved Animal Welfare or the Adverse Effects of an Opioid Drug?

**DOI:** 10.1371/journal.pone.0095283

**Published:** 2014-04-15

**Authors:** Albin Gräns, Erik Sandblom, Anders Kiessling, Michael Axelsson

**Affiliations:** 1 Department of Biological and Environmental Sciences, University of Gothenburg, Gothenburg, Sweden; 2 Department of Animal Nutrition and Management, Swedish University of Agricultural Sciences, Uppsala, Sweden; Institut National de la Recherche Agronomique (INRA), France

## Abstract

The use of fish models in biomedical research is increasing. Since behavioural and physiological consequences of surgical procedures may affect experimental results, these effects should be defined and, if possible, ameliorated. Thus, the use of post-surgical analgesia should be considered after invasive procedures also in fish, but presently, little information exists on the effects of analgesics in fish. This study assessed the effects of an opioid drug, buprenorphine (0.05 mg/kg IM), on resting ventilation and heart rates during 7 days of postsurgical recovery in rainbow trout (*Oncorhynchus mykiss*) at 10°C by non-invasively recording bioelectric potentials from the fish via electrodes in the water. Baseline ventilation and heart rates were considerably lower compared to previously reported values for rainbow trout at 10°C, possibly due to the non-invasive recording technique. Buprenorphine significantly decreased both ventilation and heart rates further, and the effects were most pronounced at 4–7 days after anaesthesia, surgical procedures and administration of the drug. Somewhat surprisingly, the same effects of buprenorphine were seen in the two control groups that had not been subject to surgery. These results indicate that the reductions in ventilation and heart rates are not caused by an analgesic effect of the drug, but may instead reflect a general sedative effect acting on both behaviour as well as e.g. central control of ventilation in fishes. This resembles what has previously been demonstrated in mammals, although the duration of the drug effect is considerably longer in this ectothermic animal. Thus, before using buprenorphine for postoperative analgesic treatment in fish, these potentially adverse effects need further characterisation.

## Introduction

The use of fish as models in bio-clinical research is increasing [1], [2]. Normally when animals are used in veterinary practice or experimental research, different forms of postsurgical analgesics are administered to alleviate postsurgical pain and speed up recovery from the surgical intervention. However, so far fish that undergo invasive procedures have not been given any form of post-surgical analgesia [1]. This is likely due to the fact that we today still know little about how fish perceive and react to pain. Although it is known that fish have nociceptors [3], the debate on whether they are actually capable of experiencing the sensation of pain is still intense [2], [4]. Nonetheless, if fish do perceive pain, an attempt to minimize this is not only important from a welfare perspective, but also desired from the researcher's point of view as it will likely increase the quality and reliability of the experimental data. In fact, it has been suggested that much historical physiological data for fish may be biased due to poor post-surgical recovery of the animals [5]–[7].

When addressing pain and pain relief in animal models it is always challenging to score and quantify the pain perceived by the animal [2], [8]. Fish models are no exception and many of the suggested indictors of pain in fish have been criticized as they may fail to separate the unconscious sensory detection of harmful stimuli from conscious pain [4]. There are, however, some physiological changes involved in the stress response in fish that are well-validated indices of fish well-being and these could potentially be used when evaluating the effects of analgesic drugs in fish. For example, in rainbow trout (*Oncorhynchus mykiss* L.), which is one of the most commonly used model species in fish physiological research, nociceptors have been identified [3] and increased ventilation rate is a well-established response to both direct nociception stimulation [3], [9], [10], as well as to other general stressors such as handling in this species [5]. While heart rate changes has not yet been clearly linked to nociception in rainbow trout, tachycardia is a common response to other stressors in fish including handling [5] and the presence of a predator [11]. Furthermore, heart rate variability (HRV), which is the variation in the time interval between heartbeats and is influenced by the activity of the autonomic nervous system, has been suggested as a powerful tool to determine the level of recovery after e.g. exercise, trauma, stress or anaesthesia in mammals. The return of normal HRV may therefore reflect the successive recovery of neuronal function also in fish [12], [13]. While HRV analysis in various fish species including rainbow trout have shown to be plausible [14], [15], practical applications of this analysis in fish to quantify e.g. stress are still in its infancy.

Physiological measurements in fish, including heart and ventilation rate, often involve some form of surgical instrumentation. This may be a problem as the surgical wounds *per se* and trailing leads will most likely cause nociception and stress reactions and affect the measured variables. In fresh water species this potential bias can be circumvent by using non-invasive methods for cardioventilatory recordings where the weak bio-potentials produced by the heart and ventilatory muscles can be recorded directly from the water by external electrodes [5], [16]. This method has previously been used successfully on several occasions in rainbow trout [5], [11].

The aim of the present study was to assess the effect of a commonly used opioid analgesic drug, buprenorphine (Temgesic, RB Pharmaceuticals, Berkshire, UK) on postoperative recovery in rainbow trout. In order to determine drug efficiency and to identify possible adverse effects we used a non-invasive method to measure ventilation, heart rate and heart rate variability; which are all believed to be important components of the stress-physiological response in fish [17]. Buprenorphine is one of the most commonly used drugs for postoperative pain relief in laboratory mammals [18], but neither the desired nor the potential adverse effects have been properly investigated in fish.

## Material and Methods

### Ethics statement

This study was performed in accordance with Swedish animal welfare laws. Animal care and experimental procedures were approved by the ethical committee of Gothenburg, ethical permit number: 143–2010. No protected species were used during the experiment.

### Experimental Animals

Rainbow trout (*Oncorhynchus mykiss*), ranging in size between 500 and 1040 g (mean±S.E.M: 743±21 g), were purchased from a local hatchery (Antens Laxodling AB, Alingsås, Sweden). The fish were held in two separate 2 m^3^ tanks supplied with aerated freshwater (10°C) from the departmental recirculating water system and fed dry trout pellets at regular intervals. The photoperiod was adjusted to 12∶12-h light-dark conditions. Upon arrival, fish were left for at least 4 weeks prior to any experimental procedures.

### Surgical procedures and experimental protocols

Animals were fasted for 7–13 days before experimental treatments. Once a week, a total of four fish (one at a time) were netted and anaesthetized in water containing 150 mg l^−1^ MS-222 (ethyl 3-aminobenzoate methanesulphonic acid, C_10_H_15_NO_5_S) buffered with 300 mg l^−1^ NaHCO_3_. They were kept in the anaesthetics until ventilatory movements ceased and weighed before being transferred to an operating table covered with water-soaked sponges. Anaesthesia was maintained by pumping aerated and buffered water containing MS-222 (75 mg l^−1^+150 mg l^−1^ NaHCO_3_) over the gills. The fish was covered with a sterile surgical drape and sterile gloves were used throughout the surgical procedures, and the surgical instruments were sterilized using a glass bead sterilizer (Simon Keller Steri 250, Burgdorf, Switzerland).

**Figure 1:**
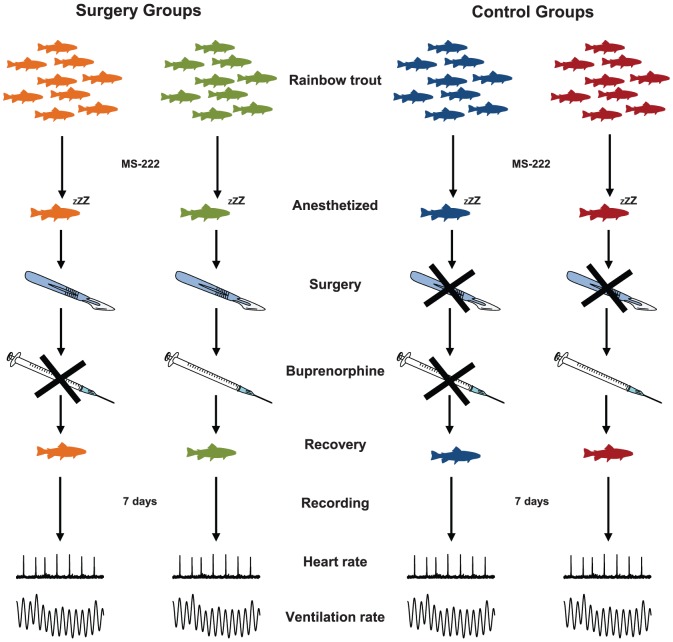
Rainbow trout (*Oncorhynchus mykiss*) divided into two experimental groups subjected to four different treatments. The “surgery groups” consist of fish in which an abdominal incision is made (indicated with a scalpel) while the “control groups” were treated identically with the only exception that no abdominal incision was made. Fish from each of the surgery and control groups were given a postoperative intramuscular injection of the opioid buprenorphine (0.05 mg kg^−1^) indicated with a syringe. After recovering from anaesthesia, ventilation and heart rates were recorded for 7 days.

On the surgery table the fish underwent one of four experimental treatments (n = 9 per treatment) as summarized in figure 1. In two fish each week a 30 mm dorsoventral incision starting at the base of the pectoral fin and running about 15 mm caudal to the operculum were made using a scalpel (‘the surgery groups’). The incisions was closed with four interrupted stitches of sterile 3/0 monofilament non-absorbable polypropylene suture (Eticon Endo-Surgery, Cincinnati, USA). This procedure was chosen to mimic a surgical technique that can be considered rather invasive and that is routinely used in rainbow trout, e.g. to access the coeliacomesenteric artery and other organs in the abdominal cavity [19]–[21]. The surgery took around 15 min, and including anaesthesia and post-operative wake up the entire procedure took around 30 min. Two additional fish each week were treated identically to the two fish in the surgery groups including netting, anaesthesia and 15 min on the surgery table. The only difference was that no abdominal incision was made in these individuals (‘the control groups’). After these experimental procedures one fish from each of the surgery and control groups were given an intramuscular injection of buprenorphine (0.05 mg kg^−1^; RB Pharmaceuticals Limited, Berkshire, UK). The order of the four treatments was randomised each week. After anaesthesia all fish were revived in fresh water and transferred to opaque experimental chambers (length: 54, width: 13 and depth: 18 cm) and an opaque plastic cover was placed over the experimental setup to minimize any external visual disturbances. The post-surgical recovery was then monitored for 7 days during which cardioventilatory activity was monitored continuously as outlined below.

### Data acquisition

**Figure 2:**
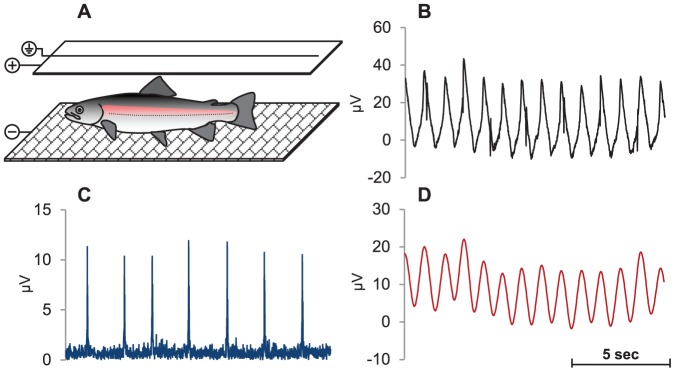
Non-invasive method for cardioventilatory recordings in fresh water. (A) Schematic illustration of Rainbow trout *(Oncorhynchus mykiss) in* the wireless recording system. (B) Example of the raw bioelectrical potential signal. (C) Example of high-pass filtered raw signal with a cut-off frequency applied at 10 Hz to identify individual heartbeats. (D) Example of low-pass filtered raw signal with a cut-off frequency at 100 Hz and smoothed with a triangular window of 201 sample points to identify ventilatory movements.

Inside each of the four experimental chambers electrodes made of stainless steel were used to detect bioelectric potentials in the water generated from the cardioventilatory muscle activity of the fish. A grid placed on the bottom of the chamber acted as one of the electrodes and a rectangle of fine stainless steel wires (2 mm diameter) placed immediately below the water surface constituted the other electrode (figure 2A). A common electric ground electrode was placed in the surrounding water. The fish could move freely between the two electrodes and the electric ground electrode without noticeable effects on the quality of the signal. The raw signals were amplified using four BIO Amplifiers (model ML136, ADInstruments, Castle Hill, Australia) and saved at a frequency of 200 Hz. The BIO Amplifier was pre-set to the following configuration: range: EEG mode, 1 mV; low-pass filter: 120 Hz; high-pass filter: 1 s and with the 50 Hz notch filter activated. The signals from the BIO Amplifiers were directed to a PowerLab 8/30 system (ADInstruments, Castle Hill, Australia) and data were collected on a PC with ADInstruments acquisition software LabChart 7 Pro v7.3.7. In LabChart, the signal was further filtered and processed off-line to separate the electrocardiogram (ECG) signal and ventilatory movements from random muscular activity (see below and figure 2 B–D).

### Data analysis

The bioelectric potentials recorded between 00∶00 and 04∶00 each day during the 7 days of recording where used in all subsequent analysis of cardioventilatory activity because during this period the spontaneous activity of the rainbow trout was found to be lowest and the room was totally dark precluding any visual stimuli. To analyse ventilation rate, heart rate and heart rate variability the HRV-module in LabChart was used to determine R-R intervals (i.e. peak-to-peak intervals). Each of the three variables were analysed over the entire 4 h recording period each day. From the filtered ECG signal (see figure 2 A), the mean heart rate and HRV was calculated. For each fish, the ECG-peaks where detected as the maximum threshold after a given threshold and with a retrigger delay of 0.5 s. This threshold value varied between 2–10 µV depending on the strength of the signal. Periods of poor signal quality due to noise from e.g. spontaneous movements were excluded from the analysis by defining a “normal” heart rate range as an R-R interval range between 500–7500 ms (equivalent to 8–120 bpm). A daily mean heart rate was calculated for each fish along with the standard deviation for all the normal RR-intervals, i.e. the heart rate variability. Similarly, the mean daily ventilatory rate was derived from the ventilation signal (see figure 2 B) using a threshold of 1 µVs^-1^ and with a retrigger delay of 0.5 s. Periods of poor ventilation signals were excluded from the analysis by defining a “normal” ventilation intervals (here analogues to the “RR-interval”) between 500–2500 ms (equivalent to 24–120 ventilations per minute; vpm). Due to considerable individual variability in signal strength, rigorous manual inspections of the detections appearing in the fringe of the data set were made to ensure that no peaks where missed or that noise was detected as peaks in the respective ventilation and ECG traces. Consequently, this resulted in a maximal number of “normal” heart and ventilation beats of >90% for all individuals.

### Statistics

All data are presented as means ± S.E.M. unless otherwise stated. Statistical analyses were conducted in SPSS 20 for Windows (SPSS Inc., Chicago, IL, USA). A linear mixed model was used for comparisons between and within treatments. Individuals were set as subjects and time (i.e. the seven days of data collection) as repeated measures. AR(1) was used as type of repeated covariance because recordings that were close in time also were more dependent than more temporally distant recordings. Heart rate, heart rate variability and ventilation rate were set as dependent variables. With or without surgical procedure and/or administration of buprenorphine together with time was included in the model as possible explanatory factors. No differences in body mass were found between experimental treatment groups or between holding tanks, and so these factors were excluded from the statistical analyses. The explanatory factors and their interactions were compared using a Sidak confidence-interval adjustment. Differences where p<0.05 were regarded as statistically significant.

## Results

**Figure 3:**
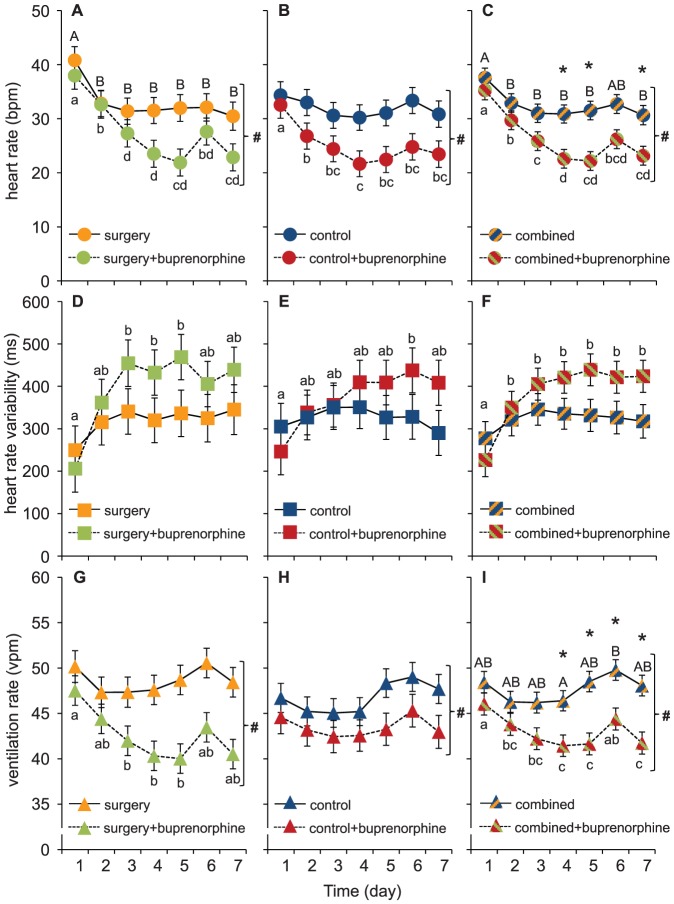
Cardioventilatory variables in rainbow trout (*Oncorhynchus mykiss*) at 10°C (*n* = 9). Data for heart rate (A–C), heart rate variability (D–F) and ventilation rate (G–I) is presented as means ±S.E.M. Different lower case letters indicate significant differences between days for the fish given an injection of buprenorphine (0.05 mg kg^−1^) and different capital letters indicate significant differences between days for the fish not given an injection of buprenorphine. # indicates a general significant effect of the drug across all 7 days and * indicates a significant difference between the groups at a specific day.

All cardioventilatory variables varied substantially, both among individuals and within individuals over time. The general trend in both ventilation and heart rate was a gradual decrease during the first 3–5 days, which levelled off or increased again on days 6 and 7 (see figure 3 A–B, G–H). The overall temporal heart rate pattern was also mirrored in the HRV response, which gradually increased during the first 3-5 days (see figure 3D–E).

Buprenorphine significantly decreased both ventilation and heart rates and the effects were most pronounced at 4–5 days after the anaesthesia, surgical procedures and administration of the drug (see figure 3 A–B, G–H). Surprisingly, the abdominal incision did not significantly affect any of the measured variables and consequently the effects of buprenorphine were seen also in the control group that had not been subjected to surgery prior to drug administration (3B, E and H). However, no significant effects of buprenorphine was found on HRV where the variation in the data was much larger compared to heart and ventilation rate (figure 3).

Since there was no significant effect of the abdominal incision, the data from both treatment groups were combined to strengthen the analysis and further explore the temporal dynamics effect of buprenorphine (see figure 3 C,F,I). In addition to the general depressant effects of buprenorphine on ventilation and heart rates determined for the individual treatment groups, these analysis uncovered a significantly depressed heart rate on days 4, 5 and 7 (fig. 3 C) and a significantly depressed ventilation rate on days 4–7 (fig. 3 I) in fish treated with the analgesic.

**Figure 4:**
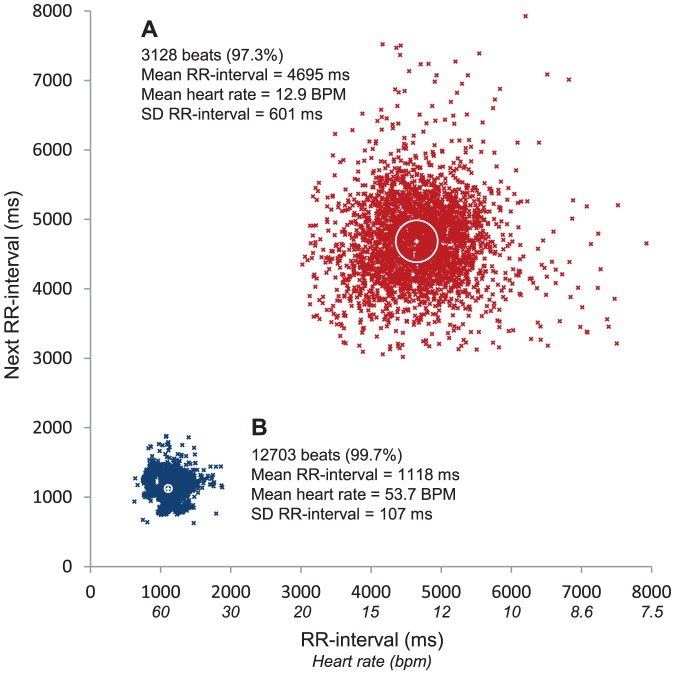
Examples of 4 hour heart rate recordings from two different fish from the control groups. (A) The fifth recording day after anaesthesia in a fish that received an injection of buprenorphine. (B) The first recording day after anaesthesia in a fish that did not receive an injection of buprenorphine. The white cross in the centre of each cloud indicates the mean heart rate for the 4 hour recording period and the white circle indicates the heart rate variability (HRV).

To illustrate the inter-individual variability and effect of buprenorphine, figure 4 shows two 4 h recordings from two individual control fish displaying extreme patterns. Fig. 4A, show all heart beats on the fifth day after treatment in a control fish injected with buprenorphine. The mean heart rate in this case was 12.9 bpm and the HRV was 601 ms. Figure 4B is from a control fish on the first day after surgery that has not been injected with buprenorphine. For this fish the mean heart rate was 53.7 bpm and consequently the HRV was only 107 ms.

## Discussion

To the best of our knowledge this is the first study exploring the effects of an opioid analgesic (buprenorphine) on cardioventilatory variables in fish. With the use of a non-invasive method we recorded heart and ventilation rates in rainbow trout for 7 days following a standardized surgical procedure. We show that buprenorphine significantly decreased both heart and ventilation rates and that the effects were accentuated during the first 4 days following surgery and/or anaesthesia and handling. However, a similar decline in cardioventilatory activity was found also in the control group without the abdominal incision, indicating that the decline in heart and ventilation rates are not analgesic effects, but may instead result from an adverse sedative effect of the opioid drug.

### Cardioventilatory actions of buprenorphine

Veterinarians and ethical boards sometimes recommend researchers to use post-surgical pain relief protocols also in fish. These recommendations are mostly based on information from mammalian studies. While this may be understandable given the limited knowledge about the effects of postoperative analgesic protocols in fish, such advices appear premature as virtually nothing is known about potential adverse effects that may interfere with experimental results. The most commonly used drug for pain relief in laboratory mammals is buprenorphine [22]. The three target genes of buprenorphine (OPRM1, OPRK1 and OPRD1) are conserved in fish (www.drugbank.ca), but there is no data available to confirm any analgesic effect in fish. In a short term experiment (3 h) on rainbow trout it was concluded that buprenorphine had minimal analgesic or anti-nociceptive actions [23], but unfortunately, such short-term studies may have limited relevance when evaluating the use of analgesic substances for postoperative treatment in fish, and the risk of masking the effects of the drug with general effects of handling stress appear to be considerable [5]. In fact, the depression in heart and ventilation rates observed in the present study using buprenorphine has been documented as adverse effects also in mammals [24]–[26], but was not found in the previous short-term study on rainbow trout [23]. In mammals, ventilatory depression is the most serious adverse effect of opioids and is caused by a direct inhibition of the respiratory centre of the brainstems [26].

Somewhat surprisingly, the same effects of buprenorphine were also seen in the fish that had no abdominal incision suggesting that the observed response is not an analgesic effect. Thus, one possibility is that the reduced cardioventilatory activity is the result of reduced spontaneous activity of the fish. In fact, a reduced activity was observed in the previous study on rainbow trout [23] and reduced activity could result from a sedative action, which is a well-known adverse effect of buprenorphine in mammals [8], [22], [25]. An alternative explanation, however, is that the sedative effect of the drug eases the stress induced by the restricted space available during this type of experiments. If this is the case the observed effects could in fact be perceived as desirable as the drug could help minimize the inevitable stress from laboratory confinement. It is also possible that the drug may interfere directly with central nervous functions regulating cardio- ventilatory control. Thus further studies are clearly needed to untangle all of the underlying behavioural and physiological mechanisms behind the cardioventilatory effects of buprenorphine in fish. Heart rate telemetry of free-swimming fish could help resolve the potential interacting effects of confinement stress and activity.

### Temporal dynamics of buprenorphine

Another somewhat surprising result was the long duration of the cardioventilatory effects induced by buprenorphine. In mammals one of the reasons for its popularity is its “long duration” of actions [18], [22], which for an opioid painkiller in mammals means half-lives in the range of a few hours in mice up to approximately one day in humans [22], [27]. In our study, however, the cardioventilatory effects of the drug were still present on day 7 after anaesthesia and drug administration. This pronounced difference is likely due to the much lower metabolic rate of ectothermic animals compared to mammals and the lower body temperature (i.e. 10°C in the trout in the present study vs. 37–40°C in mammals). Unfortunately, there is no data available on the pharmacokinetics of buprenorphine in fish, but the limited work on the pharmacokinetics of morphine (another opioid drug) estimates that the effects of a single dose administered to rainbow trout could at least in theory last well beyond a week [28].

A peculiar finding was that in the majority of the fish that were given buprenorphine there was an increase in both heart and ventilation rates on day 6 (75% of the fish increased their heart rate and 83% increased their ventilation rate). This pattern was also present in many of the control fish (50 and 61% for heart and ventilation rates, respectively). As both position of treatments in the experimental set up, as well as the weekly starting day of the experiment was randomized, we find it unlikely that this response was due to some unidentified disturbance in the laboratory environment. Instead, it is tempting to speculate that this reflected the pharmacokinetics of buprenorphine and that the depressant effect of the drug had started to decline on day 6. In mammals it is known that several metabolites of buprenorphine are biologically active and that their plasma concentrations may exceed beyond that of the parent drug [29]. A possible explanation for the shifting pattern on day 6 could therefore be that one or several metabolites of buprenorphine increase the activity level of the fish. A sudden increase in activity from fish given buprenorphine may then have disturbed the fish in the control groups as all four treatments were run simultaneously in the same experimental setup, which could explain the similar but somewhat weaker pattern in the control groups.

### Heart rate variability as a complement to heart and ventilation rate

Physiological variables in scientific publications are normally expressed as mean values with an additional indicator of variance between individuals. This is done to focus on the general tendencies of the populations/treatments. However, presenting and comparing data this way is also somewhat deceitful because the mean value inevitably ignores the intrinsic variation around the mean, which may be highly biologically relevant [14], [30]. Especially HRV has been suggested to contain valuable information on animal welfare. It is known that changes in HRV maybe a first sign of distress that can sometimes be observed when other physiological variables are still within "normal" ranges [12], [14], [31]. Heart rate variability can also be used to determine the level of recovery following e.g. exercise, trauma, stress or anaesthesia as the return of normal HRV is believed to reflect the successive recovery of neuronal function [12], [13]. The above mentioned applications of HRV to quantify stress have also been suggested to be useful in teleosts [12] but so far experimental studies in fish are rare.

In the present study we expected HRV to work as a compliment to heart and ventilation rates providing additional information about the physiological status of the fish. We also expected HRV to increase during recovery from surgery and anaesthesia, as has been demonstrated in shorthorn sculpin (M*yoxocephalus scorpius*) [12]. However, overall our findings gave little support for HRV as a superior tool to monitor the recovery process in rainbow trout. In fact, instead of providing additional information the attained HRV values were merely a noisy reflection of the mean heart rate suggesting that the latter may be a more robust indicator of post-surgical recovery in rainbow trout.

### Effects of non-invasive recording techniques, recovery time and surgical stress

The method of using submersed external electrodes was first introduced by Goodman & Weinberger (1971). Despite the obvious benefits of a method that allows cardioventilatory recordings without associated stress from surgery and handling, the method has received surprisingly limited attention in fish physiological research [5], [32]. When using this non-invasive method in combination with a recovery period of at least 3 days in rainbow trout it has been demonstrated that both heart and ventilation rates are consistently lower compared with values reported in most earlier work using surgically instrumented animals [5]. Indeed, when reviewing the current literature on cardioventilatory data for similar sized rainbow trout at ∼10°C it is obvious that our values are considerably lower than most previous values. For example, the mean heart rate of the untreated control group on day 5 in the present study (30.2±2.4 beat min^−1^) is 13–26 beats min^−1^ lower than most values reported in the literature using appropriate techniques and a post-surgical recovery time of at least 24 h [15], [21], [33]–[35]. The already remarkably low heart and ventilation rates were further reduced after an administration of buprenorphine, resulting in values 21.9±2.6 beat min^−1^ and 40.0±1.7 breaths min^−1^, respectively, which to our knowledge, are the lowest values ever reported for rainbow trout at 10°C. Nonetheless, whether this reflects a direct physiological effect of the drug or is caused by reduced spontaneous activity or reduced confinement stress remains to be shown.

As no significant effect of the surgical abdominal incision was found for any of the measured variables, part of the explanation for the unusually low heart and ventilation rates is that the fish are not instrumented with leads or catheters, and therefore not physically tethered to the recording equipment. The prolonged recovery period might also be an important reason to the low resting values, although this effect is most pronounced in fish that were given an injection of buprenorphine. Nonetheless, the importance of post-surgical recovery times beyond 24 h, which is a common benchmark in most cardioventilatory studies of fish has been emphasized before [5], [20], yet is still often ignored due to practical and logistical limitations.

## Conclusions

The present study highlights two important issues in experimental biology. First it highlights the importance of choosing an appropriate method to collect the data. When using a non-invasive method in combination with a prolonged recovery period both ventilation and heart rates are considerably lower than what has been reported in earlier studies of rainbow trout at 10°C. It is possible that our results would have been hidden by a general stress response if traditional experimental protocols had been used. Second, the importance of proper experimental design is highlighted. If we had only included the two “surgery groups” in our experimental design the conclusions would have been that the cardioventilatory effects were due to the analgesic effect of the drug. This conclusion would not have appeared strange considering these are the effects seen in mammalian laboratory animals. With two additional “control groups” the results instead revealed that the reductions in ventilation and heart rates were not caused by an analgesic effect of buprenorphine, but may instead be an adverse sedative effect of the opioid drug. Thus, before applying buprenorphine as an analgesic for fish in future biomedical research, these potential side effects need to be further characterised.
